# HTRX: an R package for learning non-contiguous haplotypes associated with a phenotype

**DOI:** 10.1093/bioadv/vbad038

**Published:** 2023-03-23

**Authors:** Yaoling Yang, Daniel John Lawson

**Affiliations:** Department of Statistical Science, School of Mathematics, University of Bristol, Bristol BS8 1UG, UK; Integrative Epidemiology Unit, Population Health Sciences, Bristol Medical School, University of Bristol, Bristol BS8 2BN, UK; Department of Statistical Science, School of Mathematics, University of Bristol, Bristol BS8 1UG, UK; Integrative Epidemiology Unit, Population Health Sciences, Bristol Medical School, University of Bristol, Bristol BS8 2BN, UK

## Abstract

**Summary:**

Haplotype Trend Regression with eXtra flexibility (HTRX) is an R package to learn sets of interacting features that explain variance in a phenotype. Genome-wide association studies (GWAS) have identified thousands of single nucleotide polymorphisms (SNPs) associated with complex traits and diseases, but finding the true causal signal from a high linkage disequilibrium block is challenging. We focus on the simpler task of quantifying the total variance explainable not just with main effects but also interactions and tagging, using haplotype-based associations. HTRX identifies haplotypes composed of non-contiguous SNPs associated with a phenotype and can naturally be performed on regions with a GWAS hit before or after fine-mapping. To reduce the space and computational complexity when investigating many features, we constrain the search by growing good feature sets using ‘Cumulative HTRX’, and limit the maximum complexity of a feature set. As the computational time scales linearly with the number of SNPs, HTRX has the potential to be applied to large chromosome regions.

**Availability and implementation:**

HTRX is implemented in R and is available under GPL-3 licence from CRAN (https://cran.r-project.org/web/packages/HTRX/readme/README.html). The development version is maintained on GitHub (https://github.com/YaolingYang/HTRX).

**Contact:**

yaoling.yang@bristol.ac.uk

**Supplementary information:**

Supplementary data are available at *Bioinformatics Advances* online.

## 1 Introduction

Numerous single nucleotide polymorphisms (SNPs) associated with human complex traits and diseases have been discovered by genome-wide association studies (GWAS) (Buniello *et al.*, 2019). Strategies to manage GWAS results, i.e. include one or multiple SNPs in a region, are realized either entirely by linkage disequilibrium (LD) considerations (Yang *et al.*, 2012) or through fine-mapping (Spain and Barrett, 2015). Haplotype-based association studies, incorporating LD information and combining gene–gene interaction into features, have the potential to be more powerful than methods based on independent SNPs (Balliu *et al.*, 2019).

Haplotype-based analysis, such as Haplotype Trend Regression (HTR) (Zaykin *et al.*, 2002) [reviewed by Schaid (2004) and Liu *et al.* (2008)], is limited to investigating haplotypes, which interact between all the specified SNPs, and they lose power if some of the SNPs do not have interaction effects. We recently (Barrie *et al.*, 2022) proposed Haplotype Trend Regression with eXtra flexibility (HTRX), which searches non-contiguous haplotypes, including single SNP effects. As the number of haplotypes increases exponentially with the number of SNPs, inferring true interactions at scale is unrealistic (Guan and Stephens, 2011). Consequently, the goal of HTRX is to make good predictions by selecting features, which have the best predictive performance. By estimating the out-of-sample variance explained (*R*^2^), HTRX quantifies whether a tagging SNP is adequate, or whether interactions or LD with unobserved causal SNPs are present. Further, by treating out-of-sample *R*^2^ as an unbiased target of inference rather than a performance measure, we provide a lower bound on the predictive power gained by haplotypes compared to SNPs alone, informing the search for specific SNP interactions.

Barrie *et al.* (2022) demonstrated the utility of this method by detecting interactions between fine-mapped SNPs in the human leukocyte antigen locus for Multiple Sclerosis. This note addresses two important improvements: controlling computational complexity, and ensuring that overfitting is controlled. We will control the former by limiting the flexibility of haplotypes to be considered, and the latter using penalization and cross-validation (CV). In addition to Bayesian Information Criteria (BIC) (Schwarz, 1978) employed to select candidate models by Barrie *et al.* (2022), we incorporate other popular methods including Akaike’s information criterion (AIC) (Akaike, 1974) and least absolute shrinkage and selection operator (lasso) (Tibshirani, 1996) regularization in the R package ‘HTRX’.

## 2 Methods

HTRX defines a template for each haplotype using the combination of ‘0’, ‘1’ and ‘X’, which represent the reference allele, alternative allele and either of the alleles, respectively, at each SNP. For example, a four-SNP haplotype ‘1XX0’ only refers to the interaction between the first and the fourth SNP. Each haplotype *H_ij_* takes a value of 0, 0.5 or 1 if a diploid sample *i* has 0, 1 or 2 copies of haplotype *j*. This template creates $3^{u}-1$ different haplotypes in a region containing *u* SNPs, while only $2^{u}-1$ of them are independent. The models and methods that we quantify the variance explained are introduced in Supplementary Method S1.1.

Fitting models using all possible haplotypes which we refer to as ‘Direct-Fit’ result in overfitting (Hawkins, 2004). To address this, HTRX considers AIC, BIC and lasso penalization. AIC and BIC penalize the number of features in the model through forward regression, while lasso uses L1 norm to regularize parameters, and retains only the features whose parameters do not shrink to 0. Model performance is evaluated using the out-of-sample variance explained by haplotypes within a region, which assesses the model’s ability to generalize to new datasets. To evaluate the model’s predictive ability, we use *k*-fold CV, an ensemble learning method (k≥3 for all the algorithms below) as a natural score function:

Algorithm 1 Direct CV (function ‘do_direct_cv’)

Generate haplotype features;

Split data into *k* folds;

**for** *i* ← 1 to *k* **do**

 Train in *k –* 1 folds (i′≠i) using penalization;

 Test in fold *i*, compute $R_{i}^{2}$; ▹ *Out-of-sample R^2^*

Compute score: $R_{\text{cv}}^{2}\;\leftarrow \;\frac{1}{k}\sum_{i=1}^{k}R_{i}^{2}$.

In Algorithm 1 and throughout, we use a linear regression or logistic regression model for training and testing. Other models could be applied to HTRX features as they are applied to SNP-based features. The ‘Generate haplotype features’ step involves enumerating the possible templates.

Although Algorithm 1 reduces overfitting, it generates an ensemble of feature sets due to variation in the training data. To choose a fixed feature set, a more complicated ‘Two-stage CV’ algorithm (Algorithm 2) is required. In the first stage, we generate a set of candidate models using penalization through simulations, the number of which is denoted by *B*. For each simulation, a fraction *D* of the available data is sampled for model training, and a total of *q* candidate models are selected. Following the simulations, we count the number of unique candidate models selected, denoted by *z*, which are subsequently passed to the second stage for the evaluation of their out-of-sample performance via k-fold CV.

Algorithm 2 Two-stage CV (function ‘do_cv’)

Generate haplotype features;

**for** *b* ← 1 to *B* **do** ▹ *Stage 1: Generate candidate models*

 Sample a fraction of data *D*;

 Select the best *q* candidate models using penalization;

Count the number of different candidate models *z*;

Split data into *k* folds; ▹ *Stage 2: Model fit via k-fold CV*

**for** *i* ← 1 to *k* **do**

 **for** *j* ← 1 to *z* **do**

  Train model *j* in *k –* 2 folds, without penalization;

  Validate in one fold, compute $R_{\mathrm{vij}}^{2}$;

  Test in remaining one fold, compute $R_{\mathrm{tij}}^{2}$;

Compute ${\bar{R}}_{vj}^{2}$←$\frac{1}{k}\sum_{i=1}^{k}R_{\mathrm{vij}}^{2}$; so $j^{*}$←$\underset{j}{\arg\;\max}{\bar{R}}_{vj}^{2}$;

Compute ${\bar{R}}_{\text{2-stage}}^{2}\leftarrow \;\frac{1}{k}\sum_{i=1}^{k}R_{tij^{*}}^{2}$.

We provide a rigorous justification of the validity of Algorithm 2 in Supplementary Method S1.2. In addition, we demonstrate that the within-CV average out-of-sample *R*^2^, as adopted by the ‘Two-stage CV’ approach, accurately estimates the out-of-sample *R*^2^ performance when tested on a completely independent dataset. However, this algorithm scales badly with *u* and is limited to around u≤6.

To consider more features as commonly found in genetic LD blocks, ‘Cumulative HTRX’ (Algorithm 3) controls the space and computational complexity. In specific, ‘Cumulative HTRX’ extends ‘Two-stage CV’ by initially sampling *L* out of the total *u* SNPs at each simulation, instead of generating the complete set of possible templates. From the templates generated exhaustively using *L* SNPs, the best *M* features are identified through a forward regression approach without penalization, which aims at retaining more ‘suffix’ of longer haplotypes. Subsequently, another SNP is sampled to create 3M+2 possible haplotypes, and the top *M* features are selected through another forward regression with no penalization. Once all SNPs have been accounted for by repeating the above process, penalization is employed to choose the best *q* models. After simulating *B* times, the optimal model is determined using a *k*-fold CV process similar to that of Algorithm 2, and its out-of-sample performance is evaluated.

Algorithm 3 Cumulative HTRX (function ‘do_cumulative_htrx’)

**for** *i* ← 1 to *B* **do** ▹ *Stage 1: Extend haplotypes*

 Sample a fraction of data *D*;

 Sample *L* features (from *u*) to generate all possible haplotypes;

 Retain best *M* features using forward regression with no penalization;

 **for** *j* ←*L *+* *1 to *u –* 1 **do**

  Sample another SNP from the remaining u−j+1 to generate all possible haplotypes;

  Retain best *M* features using forward regression with no penalization;

 Add the last SNP to *M* to generate all possible haplotypes; ▹

*Stage 2: Generate a set of candidate models*

 Select the best *q* candidate models using penalization;

Count the number of different candidate models *z*;

Apply Algorithm 2 Stage 2. ▹ *Stage 3: Constrained CV*

**Fig. 1.**
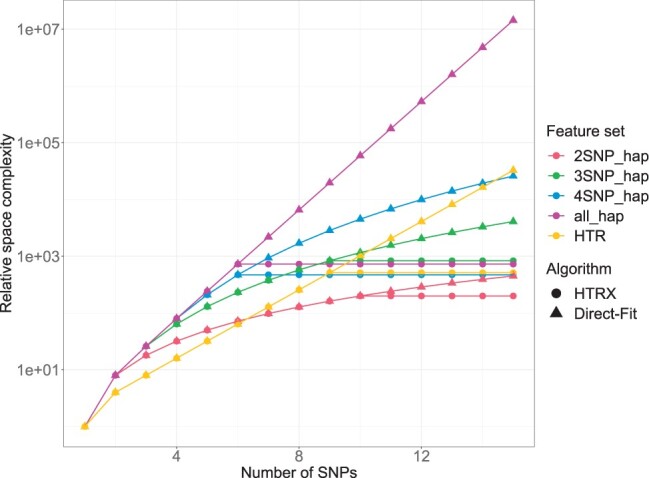
‘Cumulative HTRX’ bounds space complexity. Comparison of space complexity for Algorithm ‘Direct-Fit’ (all-feature multivariate regression) and ‘HTRX’ on different feature sets for both linear and logistic regression models. Feature set specifies the maximum number of features that can interact using ‘SNP’, ‘2SNP_hap’, ‘3SNP_hap’, etc., and ‘all_hap’ represents the all the possible haplotypes, while ‘HTR’ uses templates that interact between all features with no ‘X’ in the template. The space complexity for linear and logistic regression models is approximately proportional to the number of the input features, which is suggested to be below 1000 for ‘HTRX’. Algorithm ‘Direct-Fit’ and ‘HTRX’ begin with the same number of features L≤6. When the number of SNPs increases, the number of features increases exponentially. ‘HTRX’ uses Algorithm 3 ‘Cumulative HTRX’ to reduce the space complexity significantly. When using haplotypes with at most two or three SNPs (‘2SNP_hap’ and ‘3SNP_hap’), or all the SNPs (‘HTR’), ‘HTRX’ allows the region spanning more SNPs while keeping the relative space complexity below 1000

We refer to the HTRX Algorithm as using ‘Two-stage CV’ when L≤6 and ‘Cumulative HTRX’ for *L *>* *6. ‘Cumulative HTRX’ acts as a lower bound of the out-of-sample variance explainable by HTRX because the ‘suffix’ of significant longer haplotypes may be missed when extending haplotypes, which is the sacrifice of the considerably reduced computational cost. Figure 1 illustrates that ‘Cumulative HTRX’ brings ‘HTRX’ significant memory saving compared to ‘Direct-Fit’, which implements all-feature multivariate regression and occupies the same memory space as ‘Two-stage CV’. Larger *L*, *M* and *B* may slightly improve the predictive performance, but they significantly increase complexity both spatially and computationally.

**Fig. 2.**
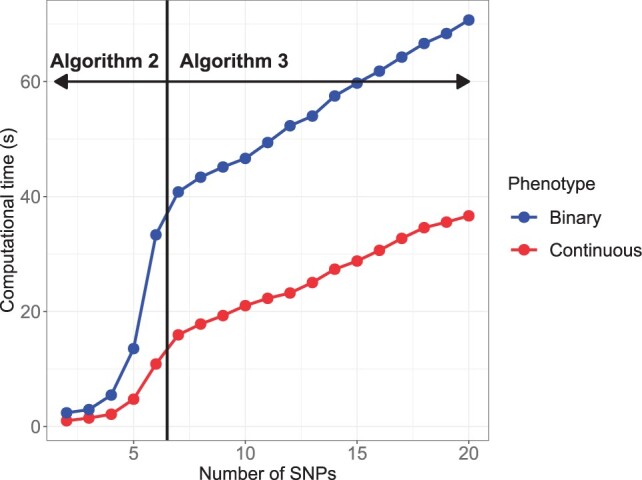
‘Cumulative HTRX’ has linear Computational Complexity. Computational time as a function of the number of SNPs, for which HTRX uses Algorithm 2 if there are no more than six SNPs and Algorithm 3 for 7–20 SNPs. Two fixed covariates are included, and the phenotype is either continuous or binary, for 2000 samples. The details are described in Supplementary Method S1.3

It is rare that many features are involved in an interaction, and such interactions are statistically hard to identify. Because of this, we consider constraining the maximum number of SNPs permitted in a haplotype template. This reduces the computational and space complexity, and enforcing a strict limit (specifically for restricting the interaction between at most two or three SNPs) enables ‘Two-stage CV’ to be performed on longer regions (Fig. 1). Additionally, we implement the comparison of the computational cost of HTRX in regions containing different number of SNPs in Supplementary Method S1.3, which indicates that ‘Cumulative HTRX’ can be applied to larger regions with only linearly increasing processing time (Fig. 2).

## 3 Results

We perform a simulation to compare the performance of different algorithms, penalization and feature sets. To generate a realistic dataset, we simulate data with the following features:

1. LD is strong within the region;
2. main SNP effects are large and sparse;
3. SNP interactions are also sparse, are weaker and may involve SNPs with and without a main effect;
4. there is confounding from an observed variable correlated with the genetic structure;
5. power is relatively low.

In detail, we simulate six biallelic SNPs *G_ij_* (i=1,…,100 000 denotes samples and j=1,…,6 denotes SNPs) with frequency about 20% for the alternative allele ‘1’ from an LD block, leading to a correlation between each pair of SNPs of around 97.8%. We assume each sample is haploid, and the effect size of two SNPs $G_{\cdot 2}$ and $G_{\cdot 4}$ are $\beta_{G_{2}}=\frac{0.5}{sd(G_{\cdot 2})}$ and $\beta_{G_{4}}=\frac{0.5}{sd(G_{\cdot 4})}$, respectively. Two haplotypes have real effects: a two-SNP interaction $H_{\cdot 1}$ (‘X0XX1X’) with effect size $\beta_{H_{1}}=\frac{0.3}{sd(H_{\cdot 1})}$ and a three-SNP interaction $H_{\cdot 2}$ (‘1XX0X1’) with effect size $\beta_{H_{2}}=\frac{0.3}{sd(H_{\cdot 2})}$. A confounder $C=0.5G_{\cdot 2}-0.8G_{\cdot 4}$ with effect size $\beta_{C}=\frac{1}{sd(C)}$, a random error with large variance $e_{i}∼N(0,4)$ and an intercept term λ=−4 are also generated.

We investigate both continuous and binary phenotypes using linear and logistic regression models. The model is: where for linear regression the phenotype *Y^c^* is continuous, simulating from $Y_{i}^{c}∼f(Y_{i}^{c})+e_{i}$. For a binary phenotype, we sample $Y_{i}^{b}∼\mathrm{Bin}(1,\pi_{i})$ where $\text{log}\;\left(\frac{\pi_{i}}{1-\pi_{i}}\right)=f(Y_{i}^{c})$.


(1) $$f(Y_{i}^{c})=\beta_{G_{2}}G_{i2}+\beta_{G_{4}}G_{i4}+\beta_{H_{1}}H_{i1}+\beta_{H_{2}}H_{i2}+\beta_{C}C_{i}+\lambda ,$$

**Fig. 3.**
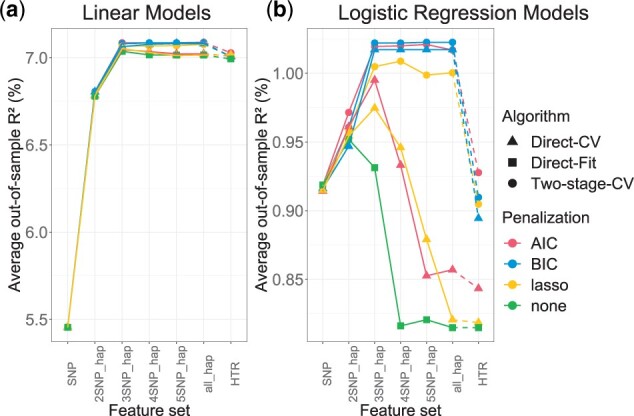
HTRX is accurate and necessary for Logistic Regression. Comparison of the average out-of-sample *R*^2^ through 10-fold CV for linear and logistic regression models in a simulated dataset. Feature set specifies the maximum number of features (of 6) that can interact using ‘SNP’, ‘2SNP_hap’, ‘3SNP_hap’, etc., and ‘all_hap’ represents the all the possible haplotypes, while ‘HTR’ uses templates that interact between all SNPs with no ‘X’ in the template. D=50%, *B* = 10 and *q* = 3 are used for ‘Two-stage-CV’ (Algorithm 2). ‘Direct-Fit’ refers to all-feature multivariate regression. ‘Direct CV’ is the implementations of Algorithm 1. (**a**) Linear Models. (**b**) Logistic Regression Models

The algorithms, penalization methods, and feature sets are then compared in terms of their out-of-sample performance under models with fixed parameters *k* = 10, D=50%, *B *=* *10 and *q* = 3 (Fig. 3). In linear models, there is little variation in out-of-sample performance regardless of the approaches above. However, for logistic regression models with binary phenotype, direct fitting the model using all features (‘Direct-Fit’) and using Algorithm ‘Direct-CV’ lead to significant overfitting, particularly when the feature set contains haplotypes with at least three SNPs (‘3SNP_hap’). This is because HTRX separates template selection as a validation step given fresh training data, which avoids overfitting, whereas ‘Direct-Fit’ does not.

The simulation for logistic regression models also reveals that under HTRX, AIC and BIC slightly outperform lasso, indicating that lasso may fail to select the correct features. Moreover, the average out-of-sample *R*^2^ reaches the maximum when the feature set includes three SNP interactions as simulated in the data. This indicates that reducing the number of allowed interactions not only speeds up computation, but may also avoid power loss. Furthermore, adding the ‘X’ to the template, i.e. enabling the search for non-contiguous haplotypes including single SNPs, increases performance, as HTRX outperforms HTR when both are selected using either ‘Direct CV’ or ‘Two-stage CV’.

## 4 Discussion

We show in a simulated dataset of six interacting features that separating validation from feature selection using ‘Two-stage CV’ significantly outperforms ‘Direct CV’, especially when the phenotype is binary (Fig. 3). Also, penalization using AIC or BIC produces significantly better out-of-sample performance than lasso. ‘Two-stage CV’ uses a validation step to select a subset of all possible models to reduce computational cost and better estimate the out-of-sample predictive performance.

Out-of-sample prediction is wasteful of scarce data, and for *R*^2^ prediction using less data creates a downward bias as well as increased variance. Whilst results throughout are shown for true out-of-sample prediction, for real data, we implement *k*-fold CV, which introduces a negligible bias compared to the variance (Supplementary Fig. S1).

‘Cumulative HTRX’ as currently implemented can be deployed on LD blocks containing all SNPs in principle, as the compute time scales linearly (Fig. 2). A natural question after conducting fine-mapping is whether some signals are lost due to interactions or the variation can totally be explained by SNPs only. It is appropriate to follow fine-mapping SNPs with a HTRX analysis to evaluate the potential for SNP interaction. Running HTRX before fine-mapping is also possible because of the linearly scaled computational cost, although power is the limiting factor in such an analysis. In both cases, it is important to remember that HTRX is limited by the power in the data, i.e. absence of evidence of interaction is not evidence of absence.

More generally, these algorithms efficiently search features for interactions. One approach is reducing the number of interactions permitted, and another is growing the most promising interaction sets. Both exploit the diminishing marginal returns of complexity for prediction to quantify the total out-of-sample variance explained, which tests for the presence of feature interaction. It has general application in regression, but is specifically important for determining whether a single SNP is an adequate description of the effect of a genetic region on a phenotype.

## Software and data availability

The source code for R package HTRX is publicly available from GitHub at: https://github.com/YaolingYang/HTRX and a tutorial for HTRX is available in the ‘vignette’ folder. The data underlying this article are available in the article and in its online supplementary material.

## Supplementary Material

vbad038_Supplementary_Data
