# Supplementary material for: HTRX: an R package for learning non-contiguous haplotypes associated with a phenotype
Source: Bioinform Adv. 2023 Mar 23;3(1):vbad038. doi: 10.1093/bioadv/vbad038 (PMC10074024; doi:10.1093/bioadv/vbad038)
Supplement: vbad038_Supplementary_Data [file vbad038_supplementary_data.zip › vbad038_Supplementary_Data/HTRX vignette-- a tutorial for using R package HTRX.pdf]

HTRX: an R package for learning non-contiguous haplotypes

Yaoling Yang, Daniel Lawson

1. Introduction

Package `HTRX` uses Haplotype Trend Regression with eXtra flexibility (HTRX) to search a pre-defined set of SNPs for haplotype patterns that include single nucleotide polymorphisms (SNPs) and non-contiguous haplotypes. We search over all possible templates which give a value for each SNP being '0' or '1', reflecting whether the reference allele of each SNP is present or absent, or an 'X' meaning either value is allowed. For example, a four-SNP haplotype '1XX0' only refers to the interaction between the first SNP's alternative allele and the fourth SNP's reference allele.

It is unrealistic to infer the true gene-gene interactions because the number of haplotypes increases exponentially with the number of SNPs. The goal of package `HTRX` is to make good predictions by selecting genetic features which have the best predictive performance. Although it is possible to use HTRX to fit the best haplotype patterns to the entire dataset, in order to avoid overfitting in the estimates of variance explained by haplotypes we instead split the dataset such that we train, select and test the model on different folds of data. By predicting the out-of-sample variance explained (R^2), HTRX quantifies whether a tagging SNP is adequate to represent a region, or whether interactions or LD with unobserved causal SNPs are present (Yang and Lawson (2022)).

Here we provide a tutorial on using the `HTRX` package for selecting haplotype models. We mainly focus on the following functions:
1. `make_htrx`: generating the feature data for haplotypes;
2. `do_cv`: selecting haplotypes for small regions;
3. `do_cumulative_htrx`: selecting haplotypes for large regions.

2. Installation

To start using the HTRX package, you need to install it from CRAN:

```
install.packages("HTRX")
```

Then you can load the package `HTRX` into R by running:

```
library(HTRX)
```

3. Data loading

First, we load three example datasets contained in the package `HTRX`.

```
## load the data
data(example_data_nosnp)
data(example_hap1)
data(example_hap2)
```

`example_data_nosnp` is the covariate data including outcome, sex, age and the first 18 ancestral Principle Components (PCs) for 5000 individuals. `HTRX` can also handle datasets with either continuous or binary

response variables. As the outcome is the binary for this example dataset, it takes values of 0 or 1 denoting having the outcome or not, respectively.

```
##
example_data_nosnp[41:43,1:6]
#>    outcome sex age        PC1        PC2         PC3
#> 41       0   1  21 -0.3889841 -0.5743902 -0.72294996
#> 42       1   1  51 -0.2182516 -0.9395326 -0.64852708
#> 43       0   0  51 -0.7374281 -0.2923432 -0.04199157
```

`example_hap1` and `example_hap2` are the genotype data (including 8 SNPs) for the first and second copy of the genomes for 5000 diploid individuals, respectively. Each SNP must be biallelic, it takes value of '0' to represent the reference allele and '1' for the alternative allele.

```
head(example_hap1,3)
#>   SNP1 SNP2 SNP3 SNP4 SNP5 SNP6 SNP7 SNP8
#> 1    0    0    1    1    0    0    0    1
#> 2    1    1    0    0    0    0    1    1
#> 3    1    0    1    1    0    0    1    1
```

4. Haplotype selection within small regions

As defined by our template, there are $3^m - 1$ haplotypes (including SNPs, continuous haplotypes and non-contiguous haplotypes) if the region contains m SNPs. This results in large memory space and computational cost when there are many SNPs included in the region. In this section, we focus on regions with **at most 6 SNPs**, while we will deal with longer regions in Section 5.

4.1 Creating haplotype data

Before performing haplotype selection within small regions, we need to represent the data in terms of haplotype templates for each individual using function `make_htrx`. In this example, we focus on the region of the first 4 SNPs for the first 2000 individuals.

```
## create haplotype data removing haplotypes rarer than 1%
HTRX_matrix_rmrare = make_htrx(hap1=example_hap1[1:2000,1:4],
                               hap2=example_hap2[1:2000,1:4],
                               rareremove=TRUE,rare_threshold=0.01)

## display the created haplotype data
HTRX_matrix_rmrare[1:3,47:50]
#>   X0X1 01X1 11X1 X1X1
#> 1  1.0    0    0    0
#> 2  0.5    0    0    0
#> 3  1.0    0    0    0

## create haplotype data without removing any haplotypes
HTRX_matrix_allhaps = make_htrx(hap1=example_hap1[1:2000,1:4],
                                hap2=example_hap2[1:2000,1:4])

## create haplotype data while at maximum 3 SNPs can interact
HTRX_matrix_3snphaps = make_htrx(hap1=example_hap1[1:2000,1:4],
                                 hap2=example_hap2[1:2000,1:4],max_int=3)

## compare the numbers of haplotypes created by setting different 'mat_int'
```

```r
cat(ncol(HTRX_matrix_rmrare),
    ncol(HTRX_matrix_allhaps),
    ncol(HTRX_matrix_3snphaps))
#> 79 80 64
```

The output of `make_htrx` is a data frame with rows denoting individuals and columns denoting haplotypes which are defined by the HTRX template. For diploid samples, such as our example, each haplotype takes a value of either 0, 0.5 or 1 if the individual has 0, 1 or 2 this haplotype in both copies of the genome. For haploid samples, only either 0 or 1 is taken for each haplotype.

There are many arguments for `make_htrx` which enables creating personalized haplotype data. For diploid samples, `hap1` and `hap2` should be the genotype data for both genomes. If the sample is haploid, we needn't specify the value of `hap2` because by default `hap2=hap1` representing haploid samples. The logical argument `rareremove` determines whether haplotypes with frequency lower than `rare_threshold` are removed, and by default `rareremove=FALSE` meaning no rare SNPs will be removed. Finally, `max_int` can be set to specify the maximum number of SNPs that can interact, but its value needn't be given if we want to select the best model from all the haplotypes. For instance, when setting `max_int=3` in the example dataset above, 16 haplotypes which are the interaction between all 4 SNPs are removed.

4.2 Selecting haplotypes

We use function `do_cv` which is a two-stage procedure to select haplotypes for regions containing at most 6 SNPs.
Stage 1: create haplotype data from SNP data, and select candidate models using AIC, BIC or lasso.
Stage 2: select the best model using k-fold cross-validation.

The details of the algorithm are described in detail in Yang and Lawson (2022).

We first explain the important arguments in `do_cv`. `data_nosnp` is a data frame whose first column must be the outcome. If the fixed covariates are included in the model, they should appear in the following columns. `featuredata` is the genetic features we will select from, which includes both non-contiguous haplotypes and SNPs. `sim_times` is the number of simulation in the step for generating candidate models. A larger `sim_times` linearly increases the computational time but is shown to improve the model performance in a very limited scale, so we suggest it takes a small value. `featurecap` limits the maximum number of features from `featuredata` to be selected. `usebinary` is determined by the type of outcome. It takes value of 1 for binary outcome and 0 for continuous outcome. We will use stratified sampling to split data if `method="stratified"` is specified. We provide three different criteria for model selection by argument `criteria`: "AIC", "BIC" and "lasso". For larger datasets, we may want to implement parallel programming by setting `runparallel=TRUE` and specifying the cores through argument `mc.cores`. Note that we use `mclapply` for parallel running, so this is currently unavailable for Windows users. The other auguments, their default values and the description can be found by typing `help(do_cv)`.

To start with the analysis, we select the best model entirely from the haplotype data `HTRX_matrix_rmrare` we created above while ignoring the sex, age and PCs for individuals. The output of `do_cv` is a list with two elements. The first element is the out-of-sample variance explained (we use standard R^2 for continuous outcome and McFadden's R^2 for binary outcome) by each fold of the test dataset, and the second element is the selected features *arranged in order of the association with the outcome.*

```r
## selecting the best haplotype model using "AIC" from all the haplotypes
CV_results_nocovar <- do_cv(data_nosnp=example_data_nosnp[1:2000,1,drop=FALSE],
                            featuredata=HTRX_matrix_rmrare,
                            sim_times=2,featurecap=4,usebinary=1,
                            method="simple",criteria="BIC",gain=FALSE)

cat('The selected features', as.character(CV_results_nocovar[[2]]),
```

```
    'explains \n',mean(CV_results_nocovar[[1]])*100,
    '% average out-of-sample variance')
#> The selected features 0XXX 01XX X000 11X1 explains
#>  54.58883 % average out-of-sample variance
```

In the actual genetics analysis, we normally include some fixed covariates into the model, e.g. sex, age and the first 18 PCs. In the below example, we add the second the third column of `example_data_nosnp` to `data_nosnp` to add the sex and age information into the model. We also set `gain=TRUE` which means we'd like to see the extra variance explained by haplotypes (details can be found in Barrie et al. (2022)).

```
## selecting the best haplotype model using "BIC" from all the haplotypes
## here we include the sex and age as fixed covariates
CV_results_withcovar <- do_cv(data_nosnp=example_data_nosnp[1:2000,1:3],
                              featuredata=HTRX_matrix_rmrare,
                              sim_times=2,featurecap=8,usebinary=1,
                              method="stratified",criteria="AIC",gain=TRUE)

cat('The selected features', as.character(CV_results_withcovar[[2]]),
    'explains \n', mean(CV_results_withcovar[[1]])*100,
    '% extra average out-of-sample variance')
#> The selected features 0XXX 00XX 11X1 1011 XX00 1X1X 1XXX explains
#>  57.18279 % extra average out-of-sample variance
```

5. Haplotype selection for large regions

Longer haplotypes are important for discovering interactions. However, there are $3^k - 1$ haplotypes in HTRX if the region contains k SNPs, making it unrealistic for regions with large numbers of SNPs. To address this issue, we proposed "cumulative HTRX" (function `do_cumulative_htrx`) that enables HTRX to run on longer haplotypes, i.e. haplotypes which include at least 7 SNPs. This is a three-stage process explains in detail by Yang and Lawson (2022):
Stage 1: extend haplotypes until all the SNPs are included.
Stage 2: generate a set of candidate models.
Stage 3: select the best model using k-fold cross-validation.

Similarly, we focus on the first 2000 individuals' data. Different from `do_cv`, the input of `do_cumulative_htrx` doesn't require the data to be represented in terms of haplotype templates. Instead, the genotype data for SNPs (`hap1` and `hap2`) are required, which are the same as function `make_htrx`. This is because we won't generate all the haplotypes at once but gradually extend haplotypes. Also, we limit the maximum number of SNPs that can interact by setting `max_int=4`.

```
## selecting the best haplotype model using "BIC"
## we include all the 8 SNPs, but specify at most 4 SNPs can interact
## we also include the sex and age as fixed covariates
cumu_CV_results <- do_cumulative_htrx(data_nosnp=example_data_nosnp[1:2000,1:3],
                                      hap1=example_hap1[1:2000,],
                                      hap2=example_hap2[1:2000,],
                                      sim_times=1,featurecap=8,usebinary=1,
                                      method="stratified",criteria="AIC",
                                      gain=TRUE,max_int=4)

cat('The selected features', as.character(cumu_CV_results[[2]]),
    'explains \n',mean(cumu_CV_results[[1]])*100,
    '% average out-of-sample variance')
#> The selected features 0XXXXXXX 01XXXXXX 1XX00X1X 100XXXX1 1X01XXX1 0X00XXX0 explains
```

```
#>  57.42831 % average out-of-sample variance
```

The output of `do_cumulative_htrx` has the same format as `do_cv`. When comparing `cumu_CV_results` with `CV_results_withcovar`, we find adding the additional 4 SNPs hardly increase the average out-of-sample variance explained, indicating these 4 SNPs are in very weak association with the outcome. This can be reflected by the first 2 selected features being just the first SNP and the interaction between first 2 SNPs.

We have avoided the exponentially increasing computational cost of `do_cv` by implementing `do_cumulative_htrx` at the cost of losing some haplotypes, but it still requires significant amount of time to run if we have large sample sets. Thus, running parallel by setting `run_parallel=TRUE` is suggested. Furthermore, we can increase the values of `sim_times`, `featurecap`, `max_int` and `L` to slightly improve the performance, but the computational cost will be increased simultaneously.

References

Yang Y, Lawson DJ. HTRX: an R package for learning non-contiguous haplotypes associated with a phenotype. bioRxiv (2022): 2022-11.29.518395.
Barrie, William, et al. "Genetic risk for Multiple Sclerosis originated in Pastoralist Steppe populations." bioRxiv (2022): 2022.09.23.509097.
