# Supplementary material for: HTRX: an R package for learning non-contiguous haplotypes associated with a phenotype
Source: Bioinform Adv. 2023 Mar 23;3(1):vbad038. doi: 10.1093/bioadv/vbad038 (PMC10074024; doi:10.1093/bioadv/vbad038)
Supplement: vbad038_Supplementary_Data [file vbad038_supplementary_data.zip › vbad038_Supplementary_Data/HTRX_SI.pdf]

1 Supplementary Methods

1.1 HTRX models

In this section, we introduce the models for Haplotype Trend Regression with eXtra flexibility (HTRX). Let Y_i denote the phenotype and C_{ic} denote the cth fixed covariate for the ith diploid sample. Let H_{ih} denote the probability of the hth haplotype for the ith sample such that

$$H_{ij} = \begin{cases} 1 & \text{if } i\text{th sample has two copies of haplotype } j, \\ 1/2 & \text{if } i\text{th sample has one copy of haplotype } j, \\ 0 & \text{otherwise.} \end{cases}$$

For haploid samples, H_{ij} takes value of either 0 or 1 representing that sample i has 0 or 1 copy of haplotype j, respectively.

We fit the linear regression model for continuous phenotype:

$$Y_i = \sum_j \beta_j H_{ij} + \sum_c \gamma_c C_{ic} + \lambda + e_i, \tag{1}$$

where λ is the intercept and e_i is the random error which follows normal distribution with mean 0. We use the standard method, i.e. the coefficient of determination, to assess the variance explained (R^2) by the model. Specifically,

$$R^2_{lm} = 1 - \frac{SS_{res}}{SS_{tot}}, \tag{2}$$

where $SS_{res} = \sum_i (y_i - f_i)^2$ is the residual sum of squares (f_i is the fitted value of the model) and $SS_{tot} = \sum_i (y_i - \bar{y})^2$ is the total sum of squares ($\bar{y}$ is the average of y_i).

For binary phenotype, Y_i takes the value of 1 for events and 0 for non-event, and $\pi_i = Pr(Y_i = 1)$ denotes the probability that the ith sample has the event. We fit the following logistic regression model:

$$\begin{aligned} &Y_i \sim \text{Bin}\left(1, \pi_i\right), \\ &\log\left(\tfrac{\pi_i}{1-\pi_i}\right) = \sum_j \beta_j H_{ij} + \sum_c \gamma_c C_{ic} + \lambda. \end{aligned} \tag{3}$$

The standard R^2 is not appropriate to estimate the variance explained by logistic regression models.

Instead, we implement McFadden's pseudo R-squared (McFadden *et al.*, 1973):

$$R^2_{glm} = 1 - \frac{\ln(L_M)}{\ln(L_0)}, \tag{4}$$

where L_M and L_0 are the likelihoods for the fitted and the null model (model with no independent variables), respectively.

The above R^2 (including R^2_{lm} and R^2_{glm}) computes the total variance explained by haplotypes $\{H_{\cdot j}\}$ and covariates $\{C_{\cdot c}\}$. We provide the flexibility to compute the extra variance explained by haplotypes only:

$$R^2(\{H_{\cdot j}\}) = R^2(\{H_{\cdot j}\} + \{C_{\cdot c}\}) - R^2(\{C_{\cdot c}\}), \tag{5}$$

which can be specified by the argument 'gain' in the function 'do_cv' and 'do_cumulative_htrx' from R package HTRX.

1.2 Algorithm remark

In this subsection, we justify the recommended algorithm 'Two-stage CV' for short regions, which naturally justifies the algorithm 'Cumulative HTRX' for longer regions. In the first stage, we sample a subset of all possible models as the candidate models, from which we select the best model through k-fold cross-validation in the second stage. Generating more candidate models can improve the estimate accuracy, which can be realized by increasing simulation times (B) or the number of best models we keep (q). Furthermore, We recommend a small percentage of sampled data (D), because sub-data with larger variation increases the possibility that different candidate models are selected.

In the second stage, we split the dataset into k folds ($k \geq 3$), and stratified sampling is applied for binary phenotype. We make a train ($k-2$ folds), validation (1 fold) and test (1 fold) data split for each time of the CV loop. The training data is used for fitting each candidate model, and the out-of-sample R^2 is computed on the validation data. Also, we record the out-of-sample R^2 computed on the test set. After repeating the process k times when each fold has been used as the validation data once, we select the best model j^* which has the maximum average out-of-sample R^2 in the validation data. We finally report the average out-of-sample R^2 of each test set, which is

independent of the training and validation set in each time of the k-fold CV, although it is used for training and validation in the other times. In principle there is a potential for this re-use of data to cause a bias. We don't split an entirely independent test data in the HTRX package, because our goal is to a) estimate out-of-sample R^2 in order to b) rank models containing interactions vs not. By averaging over k-folds, the cross-validation procedure reusing parts of the entire dataset leads to more accurate (i.e. lower root-mean-square error) estimates.

We illustrate through simulation (Fig. S1) that the within-CV average out-of-sample R^2 is a good estimator of the average out-of-sample R^2 tested on entirely independent datasets. Using the parameters in the Results section of main text and features selected from all possible haplotypes penalized by either AIC, BIC or lasso through 'Two-stage CV' for linear or logistic regression models, we compare model performance (out-of-sample R^2) in the following setups:
(1) Algorithm 2 but testing on only one fold of the CV data, as would be obtained by using a traditional training/test split;
(2) Algorithm 2 using full CV, our recommended approach for real data;
(3) Follow Algorithm 2 by training on all the CV data, but test on an independent new dataset with the same size as the CV data, which requires double the data size.

Then we compare the average and standard deviation of the out-of-sample R^2 obtained by different setups in Fig. S1. The mean values of each out-of-sample R^2 are similar, while testing on a single set of the CV data has a significantly larger standard deviation than the average of R^2 tested on each fold of the CV data. The R^2 for setup (3) is the model performance on an entirely large independent dataset while trained on all the CV data. As the training data for setup (3) is larger than (1) and (2), the fitted model should be more reliable. Compared with the R^2 for setup (3), the within-CV average out-of-sample R^2 that we report in 'Two-stage CV' and 'Cumulative HTRX' (the R^2 for setup (2)) has almost equivalent distribution for linear phenotype and minor underestimation for binary phenotype. Therefore, our estimated average out-of-sample R^2 exhibits little, if any, bias while avoiding power loss by splitting entirely independent test data.

1.3 Computational time of R package HTRX

The two main functions for package HTRX are 'do_cv' and 'do_cumulative_htrx', which correspond to 'Two-stage CV' (Algorithm 2) and 'Cumulative HTRX' (Algorithm 3), respectively. 'Two-stage CV' deals with shorter regions (at most 6 SNPs) while 'Cumulative HTRX' deals with longer regions.

As for 'Two-stage CV', the below parameters can significantly affect the computational time:
(1) B: the number of simulations in stage 1;
(2) D: the fraction of training data in stage 1;
(3) q: the number of candidate models selected by each simulation in stage 1;
(4) k: the number of folds for cross-validation in stage 2;
(5) max_int: the maximum number of SNPs that can interact.

As for 'Cumulative HTRX', there are some additional speed-related parameters to the above:
(1) L: the number of SNPs that the haplotype template of 'Cumulative HTRX' starts with;
(2) M: the number of features that 'Cumulative HTRX' retains by forward regression with no penalization when extending haplotypes.

We simulate a dataset to compare the computational time. This dataset has 20 SNPs' genotype information for 2000 samples, and the fixed covariates are sex and age. Both binary and continuous phenotypes are considered. We set $B = 1$, $D = 50\%$, $q = 3$, $k = 10$, $L = 6$, $M = 7$ and max_int is the maximum number of SNPs, and use BIC criterion for feature selection (note that criteria have little impact on the speed). We investigate regions with at least 2 and at most 20 SNPs, where 'Two-stage CV' is applied for regions with 3-6 SNPs while 'Cumulative HTRX' with 7-20 SNPs. The computational time running on an MSI laptop with an Intel Core i7-10750H processor running at 2.60GHz on a single core is shown in Fig. 2. The computational time increases slower and linearly after the region has more than 6 SNPs because 'Cumulative HTRX' can efficiently reduce the computational cost by losing just minor accuracy. This reassures that HTRX methods can be applied to investigating regions with large number of SNPs.

In addition, when performing HTRX, we provide an argument 'runparallel' in the R functions

‘do_cv’ and ‘do_cumulative_htrx’ which enable parallel programming using any number of CPUs by setting ‘mc.cores’. This can reduce the computational time T to approximately $T/$mc.cores for large datasets in practice.

Normally, fitting models using function ‘glm’ in R for binary phenotype is much slower than using function ‘lm’ for continuous phenotype. To address this, we take advantage of R package ‘fastglm’ which significantly speeds up fitting logistic regression models, and eventually the computational time for binary phenotype is controlled to be no more than twice of the continuous phenotype.

References

McFadden, D. *et al.* (1973). Conditional logit analysis of qualitative choice behavior.

2 Supplementary Figures

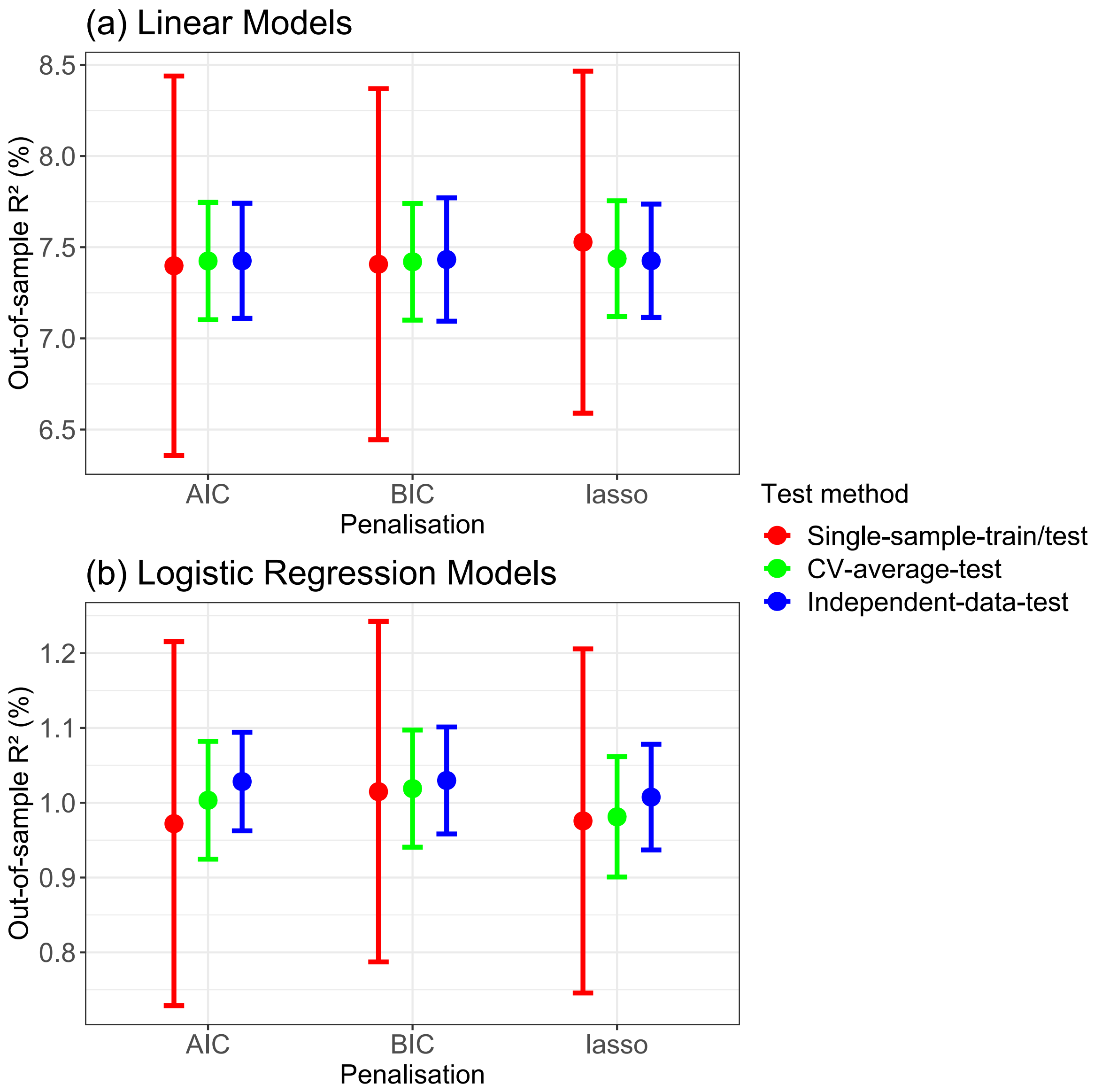

Figure S1: **Comparison of out-of-sample variance explained from different test methods.** The round dots represent the average of 250 out-of-sample R^2 from simulation, and the error bar shows the distance of one standard deviation from the average of 250 simulated out-of-sample R^2. 'Single-sample-train/test', 'CV-average-test' and 'Independent-data-test' represent different test methods specified in Supplementary Methods 1.2 (1), (2) and (3), respectively. (2) is our recommended CV approach for real data within small regions (Algorithm 2). (1) is a traditional test-train split of that data, and (3) requires additional out-of-sample data.
